# Quilt technique after mastectomy: stepped-wedge randomized cluster trial showing superior textbook outcome and reduced healthcare utilization

**DOI:** 10.1093/bjs/znaf175

**Published:** 2025-09-30

**Authors:** Lotte J van Zeelst, Joost D J Plate, Ramon R J P van Eekeren, Britt ten Wolde, Evita M A Kroeze, Emma C Schalken, Johannes H W de Wilt, Luc J A Strobbe

**Affiliations:** Canisius Wilhelmina Hospital, Department of Surgical Oncology, Nijmegen, The Netherlands; Radboudumc, Department of Surgical Oncology, Nijmegen, The Netherlands; Canisius Wilhelmina Hospital, Department of Surgical Oncology, Nijmegen, The Netherlands; Rijnstate Hospital, Department of Surgical Oncology, Arnhem, The Netherlands; Canisius Wilhelmina Hospital, Department of Surgical Oncology, Nijmegen, The Netherlands; Canisius Wilhelmina Hospital, Department of Surgical Oncology, Nijmegen, The Netherlands; Canisius Wilhelmina Hospital, Department of Surgical Oncology, Nijmegen, The Netherlands; Radboudumc, Department of Surgical Oncology, Nijmegen, The Netherlands; Canisius Wilhelmina Hospital, Department of Surgical Oncology, Nijmegen, The Netherlands

## Abstract

**Introduction:**

The quilt technique to minimize post-mastectomy seroma has been adopted slowly due to concerns about potential long-term side effects, longer operating times, and cost-effectiveness. This study aimed to evaluate the implementation of the quilt technique on textbook outcome in patients undergoing mastectomy.

**Methods:**

A stepped-wedge randomized cluster trial was conducted in 12 Dutch hospitals, each representing a cluster. Patients who underwent the quilt technique after mastectomy were compared with those with conventional closure. Primary outcome was textbook outcome, defined as the absence of wound complications, readmissions, reoperations, or unscheduled outpatient visits, and no increase in pain medication use 6 months after surgery compared to preoperative use. Secondary outcomes were related to healthcare consumption and patient satisfaction.

**Results:**

Two hundred and fifty-one patients who underwent mastectomy were included. The incidence of textbook outcome was higher in the quilted cohort compared to the non-quilted cohort, 94 of 143 (65.7%) *versus* 26 of 63 patients (41.3%, *P* = 0.003). Wound complications, including clinically significant seroma, were 24.0% in the quilted patients *versus* 55.6% in the non-quilted patients (*P* < 0.001). In the quilted cohort, 30.2% of patients required an unscheduled outpatient clinic visit, compared to 44.4% in the non-quilted cohort (*P* = 0.048). No significant differences were observed in postoperative pain, shoulder function, satisfaction and physical well-being of the chest, or cosmetic outcome.

**Conclusion:**

Quilting after mastectomy was superior to conventional closure in terms of textbook outcome and healthcare consumption without any undesirable side effects.

**Trial Registration number:**

NCT05272904, ClinicalTrials.gov.

## Teaser

In a stepped-wedge randomized cluster trial, the quilt technique after mastectomy was implemented in 12 Dutch hospitals and compared with conventional wound closure. The primary outcome was textbook outcome; secondary outcomes were related to healthcare consumption and patient satisfaction. Our findings indicate that quilting after mastectomy was superior to conventional closure in terms of textbook outcome and healthcare consumption without any undesirable side effects.

## Introduction

Although the rate of breast-conserving treatment of breast cancer is rising, mastectomy still comprises approximately 30% of all breast cancer operations in Western countries^1,2^. A common problem after mastectomy is seroma formation, with incidences reported up to 90%^3–5^. Seroma presents multifaceted challenges, including patient discomfort because of pain and tenseness, limited shoulder mobility, the need for repeated aspirations in the outpatient clinic, and delay of adjuvant therapy^3–5^. Seroma can be considered a clinically significant seroma (CSS) when aspiration is necessary to relieve pain or tension. CCS can result in complications such as surgical site infections (SSI) and is accompanied by higher rates of wound dehiscence and/or skin flap necrosis, substantially impacting postoperative recovery^6,7^. The exact pathogenesis of seroma formation remains elusive^5,8^.

Multiple surgical techniques have been explored to prevent the onset of seroma, including the use of fibrin glue, gentamicin–collagen sponges, bovine thrombin, and steroids^9–15^. A promising technique is the quilt technique, in which the dead space after surgery is minimized by fixation of the skin flaps to the underlying muscle with sutures. Previous studies have demonstrated that CSS incidence decreases significantly when the quilt technique is applied compared to the conventional closure technique^16–18^. Additional advantages of the quilt technique over the conventional technique include decreased SSIs, reduced bleeding complications, and lower healthcare consumption^6,19^. Due to eliminating a need for postoperative drains, outpatient surgery can be easily facilitated^20,21^. Moreover, a reduction in outpatient clinic visits related to complications of primary surgery, readmissions, and reoperations are reported after applying the quilt technique^7,19^. Most hospitals, however, have not adopted the quilt technique as standard practice. Concerns about increased postoperative pain, impaired shoulder function, worse cosmetic outcome, and additional operating time may partially cause this reluctance.

The aim of this stepped-wedge cluster randomized trial is to assess the implementation of the quilt technique on textbook outcome in patients undergoing mastectomy for breast cancer, as compared with the conventional method.

## Methods

### Study design

As described earlier in the prespecified and published study protocol, a stepped-wedge randomized cluster trial was designed at Canisius Wilhelmina Hospital in Nijmegen, a large peripheral teaching hospital^22,23^. The study was conducted in 12 teaching hospitals with dedicated breast centres around the Netherlands: Bernhoven Hospital (Uden), Bravis Hospital (Bergen op Zoom, Roosendaal), Catharina Hospital (Eindhoven), Diakonessenhuis (Utrecht), Hospital Gelderse Vallei (Ede), Hospital St. Jansdal (Harderwijk), Martini Hospital (Groningen), Onze Lieve Vrouwe Gasthuis (Amsterdam), Rijnstate Hospital (Arnhem), Slingeland Hospital (Doetinchem), St. Antonius Hospital (Utrecht, Nieuwegein), and Viecuri Medical Centre (Venlo). The local ethics committees of each hospital approved the study, ensuring compliance with current regulations. Initially, nine hospitals participated in the study. The quilt technique was gradually implemented in one hospital every 2 weeks after randomization for the sequence in which they started. Due to lagging inclusions, mainly caused by logistical issues (such as holiday months and understaffing), the steps were prolonged to 4 and later 8 weeks. In addition, three hospitals (Bernhoven Hospital, Slingeland Hospital, and Viecuri Medical Centre) were included in the study after the initial randomization process to ensure the criteria for sufficient power were met. These three hospitals were not randomized and began the study later than the initial cohort. Additional steps were incorporated into the study, in which two of the additional hospitals transitioned to the quilt technique. The third hospital did not transition to the quilt technique due to a substantial lag in inclusions within the conventional cohort (*Fig. S1*).

### Participants

Patients who were planned to undergo a mastectomy (including uni- and bilateral mastectomy with/without sentinel node (SN) or axillary lymph node dissection (ALND) were eligible for inclusion. Patients receiving direct breast reconstruction were excluded. Informed consent was signed. Patients were recruited between September 2022 and November 2023.

### Procedure and intervention

The quilt technique was carried out using Stratafix, with running sutures placed in 5–7 rows at a 2–3 cm distance between the stitches, as previously described^22,24^. A step-by-step written instruction and instructional videos for surgeons were used. Additionally, six participating hospitals opted for proctoring as part of the learning process: Catharina Hospital, Diakonessenhuis, Hospital Gelderse Vallei, Slingeland Hospital, St. Antonius Hospital, and Viecuri Medical Centre. The conventional technique was performed according to the local protocol of the participating hospitals, including the option to place or omit a postoperative drain.

### Study outcomes

Baseline characteristics were described in the study protocol^22^. The primary outcome, as prespecified in the protocol, was textbook outcome, reflecting an ideal surgical outcome. Textbook outcome was defined as the absence of wound complications, readmission or reoperations related to primary surgery, unscheduled visits to the outpatient clinic related to primary surgery, and no increase in postoperative use of analgesics 6 months after surgery compared with preoperative use. Outcomes were evaluated 6 months following surgery. Complications were classified using the Clavien–Dindo classification and scored if ≥1 in the Clavien–Dindo classification, that is any deviation from the normal postoperative course^25^.

Secondary outcomes included wound complications: seroma without aspiration, CSS, SSI, postoperative bleeding, wound dehiscence, or skin flap necrosis. Outcomes regarding healthcare consumption included the operating time (only unilateral mastectomy with/without SN), outpatient surgery, the number of unscheduled visits to the outpatient clinic (due to a wound problem within 6 months after surgery), and the number of readmissions and reoperations related to primary surgery.

Data were extracted from the electronic patient file and captured in the Castor electronic data capture system^26^.

Patient-reported shoulder function, pain, use of analgesics, and satisfaction and physical well-being of the chest were assessed using validated questionnaires at baseline (1–2 weeks before surgery), 2 weeks and 6 months after surgery. The Disability of Arm, Shoulder and Hand (DASH, a score from 0 to 100 was obtained; higher scores indicate worse function), Numeric Rating Scale for pain (NRS, 0–10), and the Breast Q for mastectomy, including the modules: satisfaction with breasts and physical well-being chest (a score from 0 to 100 for each module was obtained; higher scores reflect a better outcome) were used^27,28^. Furthermore, in both cohorts, cosmetic outcomes were evaluated by an independent expert panel of four female breast surgeons who were blinded to the type of surgery. They assessed standardized photos (grading as poor, fair, good, and excellent) captured at 2 weeks and 6 months after surgery.

### Power calculation

A mean difference in textbook outcome of 30% was considered clinically relevant for the sample size calculation. Based on a pilot study, textbook outcome is assumed to be 30% for conventional closure; a textbook outcome of 60% for the quilt technique was therefore considered a clinically significant improvement^19^. The sample size calculation was based on the superiority of the quilt technique. Chosen were a significance level of 0.05 and 80% power, with an intra-cluster correlation coefficient of 0.028 (based on an earlier pilot study^19^). Using the formula proposed by Hemming *et al.*^29^ for cluster randomized trials, the sample size was calculated to be 94 patients, 11 per centre. After accounting for a 20% loss-to-follow-up, the final required sample size was 113 patients, that is 13 per centre.

### Healthcare cost analysis

A cost analysis was performed based on the average costs for healthcare consumption based on the Dutch national standard in healthcare pricing^30,31^. The cost factors considered included operating time, in- *versus* outpatient surgery, and unplanned visits to the outpatient clinic. The estimated costs were €11 per minute for operating time, €8120 per outpatient surgery, €11 161 per inpatient surgery, €120 per outpatient visit, €4924 per readmission, and €4927 per reoperation^30,31^.

### Statistical analysis

The statistical analyses were performed using R software for statistical computing, version 4.1.2. with the additional package ‘lme4’ and IBM SPSS Statistics for Windows, Version 29.0^32,33^. Data were presented as median and interquartile range (i.q.r.: 25th–75th percentile) or as frequency and percentage. To assess the effect of quilting, generalized linear mixed models were used, incorporating random intercepts per centre to account for within-cluster correlations. *P* < 0.05 was considered statistically significant.

## Results

Two hundred and fifty-one patients were included in the study. Of these, 72 were in the non-quilted cohort and 179 in the quilted cohort (*Fig. S1*). Baseline characteristics are presented in *Table 1*.

**Table 1:** Baseline characteristics

Characteristics	Non-quilted cohort *n* = 72	Quilted cohort *n* = 179	*P* value
**Sex ratio (M : F)**	4 (5.6) : 68 (94.4)	2 (1.1) : 177 (98.9)	0.058
**Age (years)**	67 (58–73)	67 (54–75)	0.863
**BMI**	25 (23–30)	27 (23–31)	0.231
**Polypharmacy**	14 (19.4)	58 (32.4)	0.094
**Prior irradiation**	13 (18.1)	30 (16.9)	0.820
**NAC**	19 (26.4)	48 (26.8)	1.000
**Type of surgery**			0.459
** Unilateral mastectomy±SN**	53 (73.6)	131 (73.2)	
** Unilateral mastectomy+ALND**	4 (5.6)	11 (6.1)	
** Bilateral mastectomy±SN**	15 (20.8)	36 (20.1)	
** Bilateral mastectomy+ALND**	−	1 (0.6)	
**Postoperative drain**	38 (52.8)	2 (1.1)	<0.001
**Weight of the removed breast (g)**	695 (475–1005)	716 (497–1066)	0.758
**TNM 8 classification**			0.442
** Prophylactic**	4 (5.6)	18 (10.1)	
** ypT0N0**	2 (2.8)	8 (4.5)	
** In situ**	7 (9.7)	22 (12.4)	
** Stage I**	21 (29.2)	53 (29.8)	
** Stage II**	27 (37.5)	59 (33.1)	
** Stage III**	8 (11.1)	17 (9.6)	
** Stage IV**	2 (2.8)	1 (0.6)	

Variables are presented as median (i.q.r.: 25^th^–75^th^ percentiles) or frequency (%).

BMI, body mass index; NAC, neoadjuvant chemotherapy; SN, sentinel node; ALND, axillary lymph node dissection; TNM, Classification of Malignant Tumours.

### Textbook outcome

The primary outcome of the study, textbook outcome, was obtained in 206 patients (143 quilted and 63 non-quilted patients). Textbook outcome was higher in the quilted cohort (94 of 143 patients, 65.7%) compared to the non-quilted cohort (26 of 63 patients, 41.3%, odds ratio: 2.95, 95% c.i.: 1.45, 6.24, *P* = 0.003). The incidence of wound complications, unscheduled outpatient clinic visits, and increased analgesic usage 6 months after surgery were significantly lower in the quilted cohort than in the non-quilted cohort (*Table 2*). No significant differences were observed in textbook outcome when comparing the first half of quilted patients with the second half within each centre (data not shown).

**Table 2:** Primary outcome

Outcomes	Non-quilted cohort (*n* = 72)	Quilted cohort (*n* = 179)	*P*
**Textbook outcome**	26 (41.3)	94 (65.7)	0.003
Wound complication	40 (55.6)	43 (24.0)	<0.001
Readmission*	4 (5.6)	8 (4.5)	0.716^†^
Reoperation*	2 (2.8)	4 (2.2)	0.799^†^
Unscheduled visit to the OPC*	32 (44.4)	54 (30.2)	0.048
Increase of analgesics	8 (12.7)	1 (0.7)	0.005^†^

Variables are presented as frequency (%). OPC, outpatient clinic. ^†^The model is not robust with low numbers. *Related to primary surgery.

### Wound complications

CSS occurred significantly less often in the quilted cohort compared to the non-quilted cohort. SSI, postoperative bleeding, and wound-healing complications did not differ significantly between the cohorts (*Table 3*).

**Table 3:** Secondary outcomes regarding wound complications and healthcare consumption

	Non-quilted cohort (*n* = 72)	Quilted cohort (*n* = 179)	*P*
Seroma without aspiration	7 (9.7)	20 (11.2)	0.912
**CSS**	35 (48.6)	20 (11.2)	<0.001
Number of aspirations	3 (2–5)	3 (1–5)	
Volume of aspirations	278 (195–375)	270 (187–396)	
SSI	11 (15.3)	13 (7.3)	−†
Bleeding complication	5 (6.9)	4 (2.2)	0.167
Wound-healing complication	5 (6.9)	8 (4.5)	0.419
Other complication	2 (2.8)	6 (3.4)	0.815
Operating time (min)	75 (55–91)	82 (68–100)	0.136
Outpatient surgery	6 (8.5)	41 (23.6)	0.104
Unscheduled visits to the OPC*			0.048
0	40 (55.6)	125 (69.7)	
1–3	16 (22.2)	36 (20.2)	
>3	16 (22.2)	18 (10.1)	

Variables are presented as median (i.q.r.: 25th—75th percentile), or frequency (%). CSS, clinically significant seroma; SSI, surgical site infection; OPC, outpatient clinic. *Related to primary surgery. †Calculation of *P* is not feasible due to the limited availability of comparative data per hospital.

### Healthcare consumption

The operating time (75 min in the non-quilted cohort *versus* 82 min in the quilted cohort) and the rate of outpatient surgery (8.5% in the non-quilted cohort *versus* 23.6% in the quilted cohort) were not significantly different between the cohorts (*Table 3*). Total costs were €12.399 per non-quilted patient *versus* €11.829 per quilted patient. The quilt technique could save €570 per patient compared with the conventional method, which is 4.6% of total mastectomy costs.

### Patient satisfaction

**Fig. 1:**
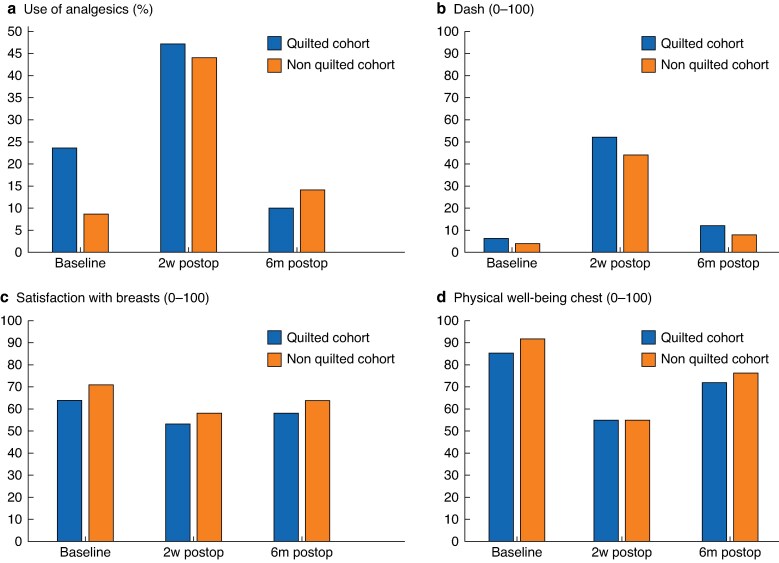
Comparison of quilted and non-quilted groups at baseline, 2 weeks, and 6 months after surgery **a** Percentage of patients using analgesics; **b** patient-reported shoulder function (DASH, higher score reflects worse outcome); **c** patient-reported Breast Q score for mastectomy, module satisfaction with breasts (0–100, higher score reflects better outcome); **d** patient-reported Breast Q score for mastectomy, module physical well-being of chest (0–100, higher score reflects better outcome). Analgesic use at baseline was significantly different (*P* = 0.009) between groups. No other statistically significant differences were observed.

Two hundred and thirty-one patients completed the questionnaire at baseline (68 non-quilted *versus* 179 quilted patients), 218 patients (65 non-quilted and 153 quilted patients) at 2 weeks after surgery, and 219 patients (67 non-quilted and 152 quilted patients) at 6 months after surgery. Patient-reported outcomes regarding postoperative pain, shoulder function, and satisfaction and physical well-being of the chest are presented in *Fig. 1*. Analgesic use at baseline was significantly higher in the quilted cohort (23.5%) compared to the non-quilted cohort (8.6%, *P* = 0.009).

### Cosmetic outcomes

**Fig. 2:**
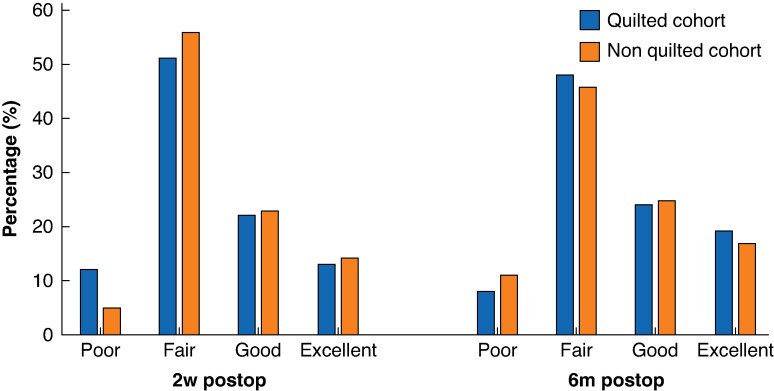
Cosmetic outcomes assessed by an expert panel using photos of the chest Outcome graded as poor, fair, good, and excellent at 2 weeks and 6 months after surgery. There were no significant differences.

Cosmetic outcomes, as assessed by the expert panel, were comparable between the two cohorts at 2 weeks (68 non-quilted *versus* 154 quilted patients) and 6 months after surgery (59 non-quilted *versus* 149 quilted patients, *Fig. 2*).

## Discussion

The impact of the quilt technique in 251 patients undergoing mastectomy for breast cancer was assessed in a stepped-wedge randomized cluster trial in 12 Dutch hospitals. The quilt technique led to a substantial improvement in textbook outcome compared to the conventional technique (65.7% *versus* 41.3%). When examining the parameters of textbook outcome, the observed difference appears to be primarily attributable to a significant reduction in CSS and a decrease in unplanned outpatient clinic visits. These findings are consistent with the hypothesis that the occurrence of complications is reduced by minimizing dead space after mastectomy, as achieved by the quilt technique, and with the results of previous studies that describe the quilt technique^6,16–19^.

The analgesic use 6 months after surgery compared to preoperative usage was significantly higher for the non-quilted cohort than the quilted cohort. However, the cohorts were too small for the model to be robust, making it difficult to draw a reliable conclusion. Moreover, the quilted group had a significantly higher analgesics use at baseline compared to the non-quilted group, and this difference seems to be due to chance. Besides the surgical technique, postoperative pain management, including perioperative field blocks or pain medication, could also play a role and was not standardized in the present study^34^.

Similar to the present study, previous studies have reported no significant differences in shoulder function, satisfaction and physical well-being of the chest, and cosmetic outcome after the quilt or conventional technique^7,19,17,35,36^. Patient satisfaction following the quilt technique after mastectomy has only been studied in the short term (up to 6 months after surgery). Studies reporting long-term outcomes are therefore needed to provide a more comprehensive understanding of patient satisfaction after the quilt technique.

In the present study, no significant difference was observed in operating time or outpatient surgery rates, although this may be due to an insufficient sample size and variability between surgeons. In literature, the use of the quilt technique has been reported to result in a 10–20 min increase in operating time, and outpatient surgery should be feasible in more than 80% of procedures^19,21,36–38^. A comprehensive protocol involving the entire surgical and anaesthetic team is essential to achieve more outpatient surgery. As outpatient surgery increases and postoperative visits to the outpatient clinic decrease after quilting, total healthcare consumption reduces^19–21^. De Rooij *et al*. conducted an economic evaluation comparing the conventional and quilt techniques and flap fixation with tissue glue after mastectomy^39^. The quilt technique was considered the most cost-effective option from a healthcare and hospital perspective. This study estimated that the quilt technique can reduce total healthcare costs by €570 per patient, based on the current costs in the Dutch healthcare system. This cost reduction could result in substantial annual savings when extrapolated to the national level. In the Netherlands, approximately 18 000 women are diagnosed with breast cancer annually, of whom almost 20% undergo a mastectomy without reconstruction^1^. Implementing the quilt technique nationwide could result in an estimated annual cost reduction of approximately €2 000 000.

The transition period in this study was 8 weeks (approximately eight patients per centre) to account for the learning curve. No significant differences were observed in textbook outcome when comparing the first half of quilted patients with the second half within each centre. This suggests that a learning curve of 8 weeks, which is relatively short, is sufficient for an experienced surgeon to adopt the quilt technique. After implementing the quilt technique, a noticeable and positive result can be expected within a short period. Post-study inquiries revealed that the quilt technique had been retained as the standard technique in nine participating breast centres, a decision motivated by the observed advantages, particularly the marked reduction in CSS formation and the relatively low incidence of associated complications. In two centres, some surgeons transitioned to the quilt technique, whereas others continued using the conventional method. In one centre, the quilt technique was not retained as a standard due to limited familiarity with the technique and the absence of an established team, as only one surgeon participated in the study.

The primary strength of the study lies in the broad-scale implementation of the quilt technique following mastectomy. Twelve teaching hospitals participated, with at least one surgeon per centre performing the quilt technique after a short transition period. Its real-world applicability accounts for the time pressures and variability in implementing clinical interventions. Using textbook outcome as a primary outcome measure makes the results robust and reproducible for most breast centres on their way to quilting. It is reassuring that this practice change can be implemented at relatively short notice, with immediately observable positive effects, and without an apparent learning curve.

A limitation of this study was the potential for bias, as neither the surgeons nor the patients were blinded to the intervention, a limitation specific to the design of pragmatic surgical clinical trials. Another limitation was that inclusions initially lagged behind schedule, resulting in a substantial imbalance in cohort sizes between the quilted and non-quilted cohorts, with a lack of patients in the non-quilted cohort. To address this, adjustments were made to the study design, resulting in deviations from the original principles of the stepped-wedge design. This could impact the interpretation of the quilting effect due to selection bias, time-related confounding, performance bias, and analysis complexity. The impact of these deviations could either favour or undermine the effects of the quilt technique, depending on factors such as the patient population and implementation conditions at the non-randomized centres. Additionally, some centres began implementing the quilt technique before their scheduled transition period, which excluded patients from the analysis. Bias might also have been introduced by baseline differences in polypharmacy and analgesic use. However, such bias would likely underestimate the true benefit of quilting, suggesting that the positive effect observed may be conservative. Moreover, the cost-effectiveness paragraph relies heavily on the situation in the Dutch healthcare system. Geographical variations in material costs, reimbursement, insurance, and logistics eventually define the amount of savings. Lastly, due to the limited number of patients undergoing axillary lymph node dissection in this study, no conclusions can be made regarding its relationship with textbook outcome in patients who undergo mastectomy and axillary lymph node dissection.

In this nationwide implementation study, quilting after mastectomy was considered superior to conventional closure in terms of textbook outcome, patient satisfaction, and healthcare consumption, with no undesirable side effects.

## Collaborators

A. Doeksen, K.C.A. van Engelenburg, M.L. Hoven-Gondrie, S.A.H. Jeuriëns-van de Ven, M.E. Keemers-Gels, A.F.T. Olieman, Y.E.A. van Riet, M.S. Schlooz-Vries, T. Schok, A.P. Schouten van der Velden, A. Smeets, M.L. Smidt, S. Vijfhuize, J.H. Volders, H.H.G. Witjes.

## Supplementary Material

znaf175_Supplementary_Data

## Data Availability

The data set generated during the current study is available from the corresponding author upon reasonable request.
