# Supplementary material for: Quilt technique after mastectomy: stepped-wedge randomized cluster trial showing superior textbook outcome and reduced healthcare utilization
Source: Br J Surg. 2025 Sep 30;112(9):znaf175. doi: 10.1093/bjs/znaf175 (PMC12483138; doi:10.1093/bjs/znaf175)
Supplement: znaf175_Supplementary_Data [file znaf175_supplementary_data.zip › Supplementary_Material.docx]

**Quilt technique after mastectomy: stepped wedge randomized cluster trial showing superior textbook outcome and reduced healthcare utilization**

**LJ van Zeelst^1,2^, JDJ Plate^1^, RRJP van Eekeren^3^, B ten Wolde^1^, EMA Kroeze^1^, EC Schalken^1^, JHW de Wilt^2^, LJA Strobbe^1^**

1 Canisius Wilhelmina Hospital, Department of Surgical Oncology

2 Radboudumc Department of Surgical Oncology

3 Rijnstate Hospital, Department of Surgical Oncology

**Corresponding author** LJ van Zeelst. Weg door Jonkerbos 100, 6532 SZ, Nijmegen, The Netherlands. [l.vanzeelst@cwz.nl](mailto:l.vanzeelst@cwz.nll.vanzeelst)

**Supplementary Materials – Index**

| **Supplementary Appendixes** |  |
| --- | --- |
| The Quilt Collaboration group | *page 3* |
| **Supplementary Figures and Tables** |  |
| Supplementary Figure 1, study timeline | *page 4* |

|  |  |
| --- | --- |

**Supplementary Appendix – The Quilt collaboration group**

A Doeksen^1^, KCA van Engelenburg^2^, ML Hoven-Gondrie^3^, SAH Jeuriëns-van de Ven^4^, ME Keemers-Gels^5^, AFT Olieman^6^, YEA van Riet^7^, MS Schlooz-Vries^5,8^, T Schok^9^, AP Schouten van der Velden^10^, A Smeets^11^, ML Smidt^12^, S Vijfhuize^13^, JH Volders^14^, HHG Witjes^15^

1 St. Antonius Hospital, Department of Surgical Oncology

2 Slingeland Hospital, Department of Surgical Oncology

3 Hospital Gelderse Vallei, Department of Surgical Oncology

4 Bernhoven Hospital, Department of Surgical Oncology

5 Canisius Wilhelmina Hospital, Department of Surgical Oncology

6 Martini Hospital, Department of Surgical Oncology

7 Catharina Hospital, Department of Surgical Oncology

8 Radboudumc Department of Surgical Oncology

9 VieCuri Medical Centre, Department of Surgical Oncology

10 St. Jansdal Hospital, Department of Surgical Oncology

11 University Hospitals Leuven, Department of Surgical Oncology

12 Maastricht UMC+, Department of Surgical oncology

13 Bravis Hospital, Department of Surgical Oncology

14 Diakonessenhuis, Department of Surgical Oncology

15 Onze Lieve Vrouwe Gasthuis, Department of Surgical oncology

**Supplementary Figure**

Supplementary Figure 1, Study timeline
