# Supplementary material for: Quilt technique after mastectomy: stepped-wedge randomized cluster trial showing superior textbook outcome and reduced healthcare utilization
Source: Br J Surg. 2025 Sep 30;112(9):znaf175. doi: 10.1093/bjs/znaf175 (PMC12483138; doi:10.1093/bjs/znaf175)
Supplement: znaf175_Supplementary_Data [file znaf175_supplementary_data.zip › CONSORT-2010-Flow-Diagram.doc]

**CONSORT 2010 Flow Diagram**

**Allocation**

**Analysis**

**Follow-Up**

**Enrollment**

Assessed for eligibility (n=938)

Excluded (n=645)

♦  Not meeting inclusion criteria

♦  Declined to participate

♦  Logistical reasons

Analysed (n=179)

Lost to follow-up (patient withdrew or died) (n=8)

Allocated to quilt technique (n=188)

♦ Received quilt technique (n=187)

♦ Did not receive quilt technique (not suitable for quilt technique) (n=1)

Lost to follow-up (patient withdrew or died) (n=7)

Allocated to conventional technique (n=105)

♦ Received conventional technique (n=79)

♦ Did not receive conventional technique (quilt technique before or during transition period) (n=26)

Analysed (n=72)

Included (n=293)
